# Assessing the intracranial metabolic score as a novel prognostic tool in primary CNS lymphoma with end of induction-chemotherapy ^18^F-FDG PET/CT and PET/MR

**DOI:** 10.1186/s40644-024-00798-1

**Published:** 2024-11-11

**Authors:** Yiwen Mo, Yongjiang Li, Yuqian Huang, Mingshi Chen, Chao Zhou, Xinling Li, Yuan Wei, Ruping Li, Wei Fan, Xu Zhang

**Affiliations:** 1 Department of Nuclear Medicine, State Key Laboratory of Oncology in South China, Collaborative Innovation Center of Cancer Medicine, Sun Yat-Sen University Cancer Center, Guangzhou, 510060 China; 2 Department of Nuclear Medicine, Sichuan Cancer Center, Sichuan Cancer Hospital and Institute, University of Electronic Science and Technology of China, Chengdu, 610041 China; 3 Department of Biomedical Engineering and Physics, Amsterdam UMC, Location University of Amsterdam, L0‑112- 1 Meibergdreef 9, Amsterdam, AZ 1105 The Netherlands; 4 Department of Radiology and Nuclear Medicine, Amsterdam UMC, Location University of Amsterdam, Amsterdam, The Netherlands; 5 Department of Nuclear Medicine, The Affiliated Cancer Hospital of Zhengzhou University & Henan Cancer Hospital, Zhengzhou, 450008 China

**Keywords:** Primary central nervous system lymphoma, PCNSL, ^18^F-FDG, PET/MR, PET/CT, Deauville score

## Abstract

**Background:**

The metabolic response of primary central nervous system lymphoma (PCNSL) patients has yet to be evaluated. This study aimed to assess the prognostic value of a novel scoring scale, the intracranial metabolic score (IMS), in PCNSL patients receiving end-of-therapy ^18^F-FDG PET/CT (EOT-PCT) and PET/MR (EOT-PMR).

**Methods:**

The IMS was determined based on the metabolism of normal intracranial structures, including gray matter, white matter, and cerebrospinal fluid. The EOT-PCT cohort was evaluated using the IMS and commonly used Deauville score (DS). Another cohort of patients who underwent the EOT-PMR was used to validate the accuracy of the IMS.

**Results:**

In total, 83 patients were included in the study (38 in PET/CT cohort, and 45 in PET/MR cohort). The area under the curve (AUC) values of the IMS for predicting PFS and OS were superior to those of the DS. When patients in the PET/CT cohort were stratified into five groups (respectively labeled IMS 1–5), three groups (IMS1-2, IMS 3–4, and IMS 5), or two groups (IMS1-3 and IMS4-5; IMS 1–4 and IMS 5), a higher IMS score was significantly correlated with poorer PFS and OS (*p* < 0.001). Similar results were observed for PFS in the PET/MR cohort (*p* < 0.001). The IMS and DS scale were found to be independent prognostic indicators for PFS and OS in the PET/CT cohort, and the IMS was identified as the sole independent prognostic indicator for PFS in the PET/MR cohort.

**Conclusion:**

The IMS as a novel and effective prognostic tool for PCNSL patients, showing superior predictive value for patients’ outcomes compared to the DS when assessed with EOT-PET scans.

**Supplementary Information:**

The online version contains supplementary material available at 10.1186/s40644-024-00798-1.

## Introduction

Primary central nervous system lymphoma (PCNSL) is a rare disease that accounts for 6% of all primary brain tumors and 4–6% of extra-nodal lymphomas, and is confined to the central nervous system (CNS) at diagnosis [1, 2]. Patients with PCNSL have a poorer prognosis, and the 5- and 10-year relative survival rates are 35.2% and 27.5%, respectively [3]. High-dose methotrexate (HD-MTX) combined with induction chemotherapy followed by consolidation therapy is currently considered the standard treatment [4]. Consolidation with HD-MTX and autologous stem cell transplantation (ASCT), chemotherapy alone, or whole-brain radiation (WBRT) reduces the risk of recurrence [5].

The response to first-line therapy is a critical predictor of prognosis and survival, making it a reliable alternative endpoint for tailoring treatment to the aggressiveness of the disease. The current standard response assessment for PCNSL imaging involves the International PCNSL Collaborative Group (IPCG) recommendations, which rely upon contrast-enhanced magnetic resonance imaging (MRI) [6]. However, its efficacy in assessing residual disease was questioned, for several studies [7, 8] have reported early detection of recurrence in patients who were in complete response (CR) on end-of-treatment (EOT) MRI. Van et al. found that there were no significant differences in outcome between patients with CR or partial response (PR) on MRI at the end of therapy [9]. Therefore, although MRI is a valuable method in the assessment of PCNSL, there is a critical need to develop a robust prognostic tool.

^18^F-fluorodeoxyglucose (FDG) positron emission tomography (PET) has been recommended as the gold standard in response assessment at the end of treatment for FDG-avid systemic lymphomas [10]. However, the utility of FDG PET in PCNSL remains an area with limited available data. Few studies [11–16] have explored FDG-PET for response assessment in PCNSL. Birsen et al. [17] found that negative interim FDG PET was associated with longer PFS. Conversely, Jo Jae-Cheol et al. [18] found that interim brain PET/CT was not associated with patient outcomes, but final PET/CT results held significant predictive value for PFS. These studies in question merely classified PET finding as either negative or positive, lacking a standardized approach for interpreting PET images.

The Deauville 5-point score (DS), which is a visual score based on comparing glucose uptake to the liver and mediastinum, is the most widely used response assessment criterion in FDG-avid systemic lymphomas [10]. Considering that the vast majority of PCNSL exhibit intense ^18^F-FDG uptake [19] at baseline, the DS response assessment scale can also be applied to patients with PCNSL. However, the role of DS in PCNSL has not been elucidated.

Hybrid PET/MR imaging combines PET and MRI during a single imaging session and could provide valuable complementary functional and anatomical information. With PCNSL localized to the brain, eye, and leptomeninges, brain PET/MR imaging is expected to be a vital means of evaluating the response in PCNSL patients in the future. However, the DS, which is currently the most commonly used score, is not applicable for brain PET/MR due to the lack of reference organs (hepatic and mediastinal blood pools).

Therefore, this study first analyzed the prognostic value of EOT ^18^F-FDG PET/CT (EOT-PCT) for progression-free survival (PFS) and overall survival (OS) in PCNSL patients using two criteria: the DS and a novel scoring system based on the background of intracranial structures that is called the intracranial metabolism scoring (IMS) system. Moreover, we applied the IMS to a cohort of PCNSL patients who underwent brain EOT PET/MR imaging (EOT-PMR) to verify its accuracy.

## Methods

### Patients

This retrospective study was approved by the ethics committees of Sun Yat-sen University Cancer Center (SYSUCC). The inclusion period ranged from January 2014 to October 2022. All patients were newly proven to be PCNSL and were referred for EOT-PCT or EOT-PMR after 6 or 8 courses of induction chemotherapy. The exclusion criteria included concomitant malignancy, the absence of EOT-PCT or EOT-PMR imaging, the interval from the completion of last chemotherapy to PET scans over 8 weeks, and leptomeningeal disease and eye involvement, which were diagnosed via CSF flow cytometry and vitreous aspirate. The patients included were divided into two cohorts (the PET/CT cohort and PET/MR cohort) according to the EOT examination. Clinical data, including age, sex, immune system condition (human immunodeficiency virus (HIV) positive or negative), Eastern Cooperative Oncology Group (ECOG) performance status, Karnofsky score (KPS), lactate dehydrogenase (LDH) level, cerebrospinal fluid (CSF) protein concentration, and pathological subtype, were collected. The International Extranodal Lymphoma Study Group (IELSG) score was calculated based on 5 factors (age > 60 years, ECOG > 1, LDH > normal, CSF > normal, and deep brain lesions) [20], and the Memorial Sloan-Kettering Cancer Center (MSKCC) score was calculated based on two factors (age > 50 years and KPS ≥ 70) [7]. The deep brain lesions included those located in the periventricular region, basal ganglia region, corpus callosum, brainstem, and cerebellum. Treatment results and follow-up data, including the dates of initial chemotherapy, PET/CT scans, PET/MRI scans, therapy regimen, disease progression, relapse or death, and follow-up data, were obtained from the SYSUCC database.

The majority of patients (81/83, 98%) received induction chemotherapy combined HDMTX-based regimen (3.5–7.0 g/m^2^, and delivered in 2–3 h intravenous infusion). The induction regimens included 35 patients (42%) in MT-R (HD-MTX, temozolomide and rituximab), 30 (36%) in MIT (HD-MTX, ibrutinib, temozolomide), 5 (6%) in MT (HD-MTX, temozolomide), and 13 (16%) in other regimens. On completion of induction chemotherapy, 23 patients (28%) received consolidation with WBRT, 16 (19%) with ASCT, and 40 patients (48%) received maintenance chemotherapy with temozolomide, zanubrutinib, obrutinib, or ibrutinib at the discretion of physician, dependent on age and comorbidities. Corticosteroids (dexamethasone is typically used) were commonly administered after biopsy.

PFS was defined as the interval between the initiation of treatment and the progression of the disease or the death of the patient. OS was calculated as the time from the initiation of treatment to the time of death from any cause.

### ^18^F-FDG PET/CT imaging

All patients fasted for six hours prior to the administration of ^18^F-FDG, and their blood glucose levels were required to be stable and lower than 200 mg/dL (11.1 mmol/L). ^18^F-FDG PET/CT scans were performed using integrated PET/CT scanners (Biograph mCT, Siemens Healthcare, Henkestr, Germany; or uEXPLORER, United Imaging Healthcare, Shanghai, China). Patients were injected with 3.7 ± 0.37 MBq (0.1 ± 0.01) per kilogram of body weight. CT scans were obtained in an arm-up position using a Biograph mCT apparatus (tube current of 80–200 mAs, voltage of 120 kV, rotation time of 0.5 s, pitch of 1.0, field of view of 50 cm, collimation of 32 × 1.25 mm, slice thickness of 3 mm) and were reconstructed in a 512 × 512 matrix. Whole-body imaging from the skull to the mid-thigh was performed in 6–8 bed positions, taking 1.5–2 min per bed with the Biograph mCT. The PET images were reconstructed with a slice thickness of 3.25 mm (2D) in a 128 × 128 matrix or 2 mm (3D) in a 200 × 200 matrix using the ordered subsets expectation maximization (OSEM) iterative image reconstruction method. For the United Imaging Health care facility, all PET images were reconstructed using an ordered subset expectation maximization algorithm with the following parameters: TOF and PSF modeling, 2 iterations and 20 subsets, a matrix of 192 × 192, a slice thickness of 2.89 mm, an FOV of 600 mm (pixel size 3.125 × 3.125 × 2.89 mm3) with a Gaussian postfilter (3 mm), and all necessary corrections, such as attenuation and scatter correction. Using these parameters to reconstruct 194-cm full-axial-length images, it took approximately 4 min after finishing PET data acquisition to generate the images for G900. All the images were transferred to a commercial medical image processing workstation (uWS-MI, United Imaging Healthcare) for image evaluation. (10.1007/s00259-020-04823-w).

### ^18^F-FDG PET/MRI

All PET/MR images were acquired using a hybrid PET/MRI system (United Imaging Medical, Shanghai, China). Patients were required to fast for six hours, and their blood glucose levels were measured before the exam. The patients were injected with 3.7 MBq/kg of FDG. Images were reconstructed into a 256 × 256 × 113 matrix with a 2.4 × 2.4 × 2.85 mm3 voxel size using the OSEM algorithm with 20 subsets and 2 iterations with the point spread function (PSF) and TOF modeling. The MRI protocols included a 3D T1-weighted spin‒echo sequence, T2-weighted perfusion, diffusion-weighted imaging (DWI), apparent diffusion coefficient (ADC) mapping, and 3D fluid-attenuated inversion recovery (FLAIR) imaging.

### Image analysis

**Fig. 1:**
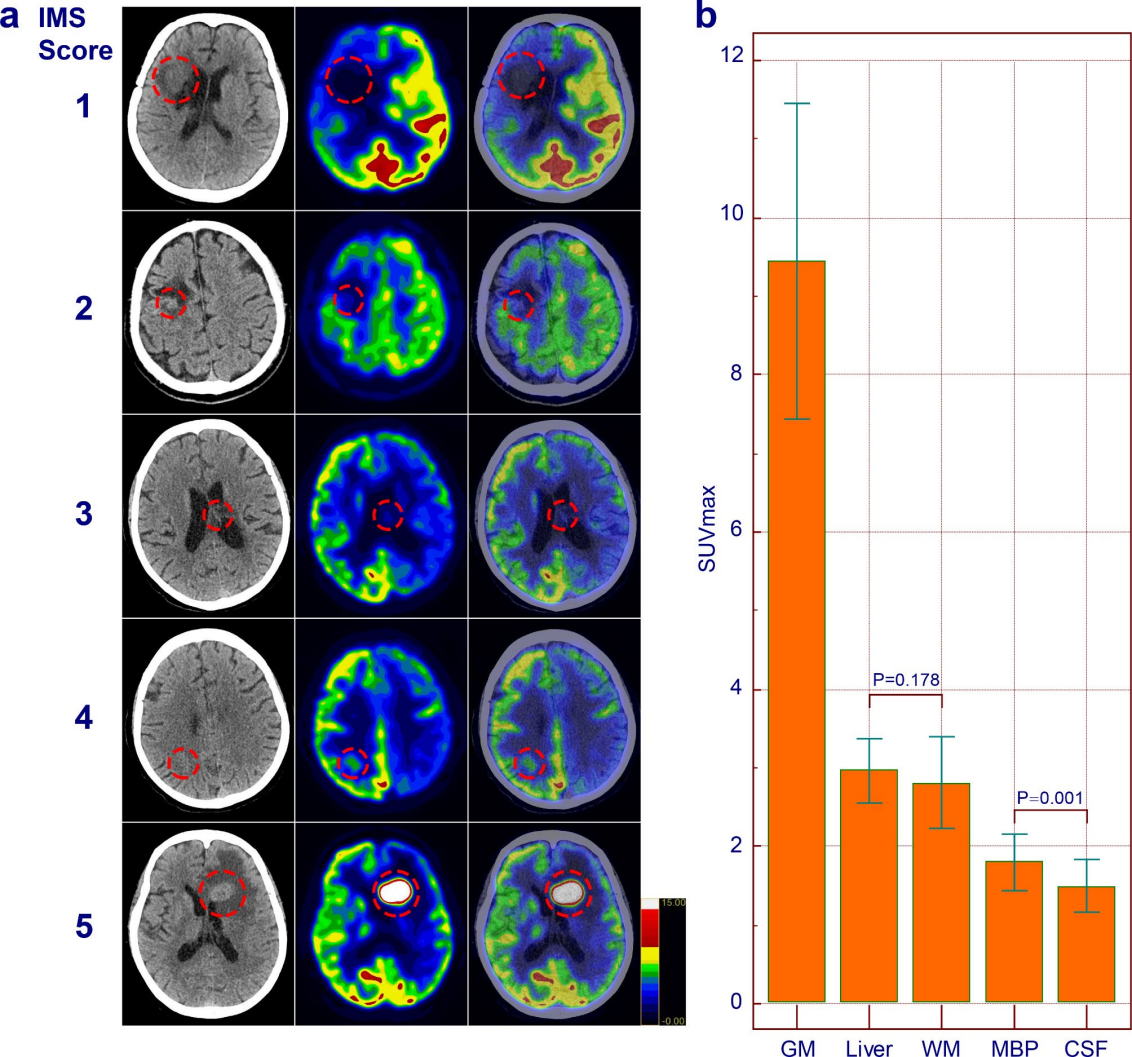
**a** Examples of the IMS scale based on FDG metabolism in normal intracranial structures (IMS 1, no residual uptake; IMS 2, residual uptake > cerebrospinal fluid but < cerebral white matter; IMS 3, residual uptake similar to cerebral white matter; IMS 4, residual uptake > cerebral white matter but < gray matter; IMS 5, residual uptake > gray matter and/or the presence of new lesions). **b** Comparison of the SUVmax of the reference normal structures for the IMS and DS scales. IMS, intracranial metabolism score; DS, Deauville score; GM, gray matter; WM, white matter; CSF, cerebrospinal fluid; MBP, mediastinum blood pool

The EOT-PCT and EOT-PMR data were reviewed blindly and independently by two experienced nuclear medicine physicians through visual and semiquantitative evaluation. The PET/CT data were analyzed using two sets of criteria. The first set of criteria was visual 5-DS, which is based on FDG uptake in the mediastinum and liver and is defined as follows: DS1 (no residual uptake), DS2 (residual uptake ≤ mediastinum), DS3 (residual uptake > mediastinum but < liver), DS4 (residual uptake moderately > liver), or DS5 (residual uptake markedly > liver, defined as SUV_lesion_ > 2 SUV_liver_ and/or the presence of new lesions). The second set of visual criteria, IMS, was based on FDG metabolism in the intracranial region and was defined as follows: 1, no residual uptake; 2, residual uptake > cerebrospinal fluid but < cerebral white matter; 3, residual uptake similar to cerebral white matter; 4, residual uptake > cerebral white matter but < gray matter; and 5, residual uptake > gray matter and/or the presence of new lesions (Fig. 1a). The EOT-PMR data were analyzed using the visual IMS. SUV is calculated by ROI activity(mCi/mL) * body weight(g)/injected dose(mCi).

For semiquantitative measurement of the reference organs, regions of interest (ROIs) were manually drawn by one observer onto the FDG-PET scans under CT or MRI guidance. The SUV_max_ of gray matter and white matter was obtained by drawing the ROI in the normal tissue of the contralateral hemisphere [21]. The ROIs of cerebrospinal fluid were placed on the lateral ventricles. The ROIs were drawn to be as large as possible and were always more than 1 cm in diameter. The liver SUV_max_ was determined by drawing a 3 cm diameter, three dimensional ROI on the normal right lobe of the liver. The mediastinal SUV_max_ was determined by drawing a 1.5 cm diameter region of interest (ROI) over contiguous slices on the descending aorta, carefully excluding the wall from the ROI. A consensus was reached for both physicians for further analysis. When there is disagreement in visual evaluation, the quantitative method is used to determine the score. We also consulted a more experienced nuclear medicine physician in lymphoma imaging if opinions are not uniform.

### Statistical analysis

The data analysis was performed using SPSS Statistics software (IBM Corp., Chicago). The basic characteristics of the PCT and PMR cohorts were compared using the chi-square test. Receiver operating characteristic (ROC) analysis was also conducted to determine the predictive accuracy of the models for 5-year PFS and OS, and the corresponding area under the curve (AUC) was calculated. Survival distributions were plotted using the Kaplan‒Meier method, and the differences were statistically evaluated using the log-rank test. Both univariate and multivariate analyses were applied using the Cox proportional hazards regression model to identify the independent prognostic indicators. The threshold for statistical significance was set at a P value of less than 0.05.

## Results

### Patient characteristics

From January 2014 to October 2022, a total of 83 patients were included in the study (38 in PET/CT cohort, and 45 in PET/MR cohort). The median intervals (25th-75th) from the completion of the last chemotherapy to PET/CT and PET/MR scans were 27 days (18–45 days) and 25 days (14–50 days), respectively.

The baseline clinical characteristics of these patients are outlined in Table 1. All patients were high-grade B-cell lymphoma, the majority of which were diffuse large B-cell lymphoma. In the PET/CT cohort, 20 patients (53%) were male, with a median age of 57 years (range 22–74). At the time of diagnosis, multiple intracranial tumor lesions were detected in 21 patients (55%), while the remaining 17 patients (45%) presented with a single lesion. In the PET/MR cohort, 24 patients (53%) were male, with a median age of 63 years (range 15–83). Multiple lesions were identified in 29 patients (64%) at diagnosis, while 16 patients (36%) had a single lesion. The median follow-up was 34.6 months in the PET/CT cohort, and the median PFS and OS were 31.2 and 38.6 months, respectively. In the PET/MR cohort, the median PFS and OS were 20.8 and 21.4 months, respectively, with a median follow-up 21.4 months. During the follow-up period, 12 patients (32%) in the PET/CT cohort experienced disease progression or relapse, and 7 patients (18%) died. In the PET/MR cohort, 8 patients (18%) experienced disease progression or relapse, and 3 patients (7%) died.

**Table 1:** Patient characteristics

Variables	PET/CT cohort (*n* = 38)	PET/MR cohort (*n* = 45)	*P*-value
Gender			
Male	20 (52.6%)	24 (53.3%)	0.949
Female	18 (47.4%)	21 (46.7%)	
Median age (range)	57 (22–74)	63 (15–83)	/
Number of tumor lesion			
Single	17 (44.7%)	16 (35.6%)	0.394
Multiple	21 (55.3%)	29 (64.4%)	
Site of tumor lesion			
Superficial	13 (34.2%)	10 (22.2%)	
Deep	11 (28.9%)	15 (33.3%)	0.476
Both	14 (36.9%)	20 (44.5%)	
Histology			
DLBLC	35 (92.1%)	44 (97.8%)	0.492
HDBLC	3 (7.9%)	1 (2.2%)	
ECOG			
0–1	19 (50.0%)	26 (57.8%)	0.479
2–3	19 (50.0%)	19 (42.2%)	
IELSG			
0–1	20 (52.6%)	14 (31.1%)	0.047
2–3	18 (47.4%)	31 (68.9%)	
MSKCC			
0	12 (31.6%)	12 (26.7%)	
1	17 (44.7%)	24 (53.3%)	0.737
2	9 (23.7%)	9 (20.0%)	
Disease Progression			
Yes	12 (31.6%)	8 (17.8%)	0.143
No	26 (68.4%)	37 (82.2%)	
Survival			
No	7 (18.4%)	3 (6.7%)	0.193
Yes	31 (81.6%)	42 (93.3%)	

*Abbreviations* DLBCL, diffuse large B cell lymphoma; HDBCL, high grade large B cell lymphoma; ECOG, Eastern Cooperative Oncology Group; IELSG, International Extra-nodal Lymphoma Study Group; MSKCC, Memorial Sloan-Kettering Cancer Center

### IMS and/or DS in the PET/CT and PET/MR cohorts

Figure 1b shows the SUV_max_ of the reference normal structures for both the IMS and DS. The SUV_max_ of gray matter, with a mean ± standard deviation (SD) of 9.45 ± 2.01, was significantly greater than that of the other reference structures. The second tier included the liver (2.96 ± 0.42) and white matter (2.81 ± 0.58), and no significant difference in the SUV_max_ was observed (*p* = 0.178). The third tier comprised the mediastinal blood pool (1.80 ± 0.36) and cerebrospinal fluid (CSF) (1.50 ± 0.34). Although the SUV_max_ of the mediastinal blood pool was significantly greater than that of the cerebrospinal fluid (*p* = 0.001), the difference in values was minimal (1.80 vs. 1.50).

Within the cohort of 38 patients undergoing EOT-PCTs, the distribution of the IMS was as follows: 5 patients (13%) were assigned IMS 5, 5 patients (13%) IMS 4, 11 patients (29%) IMS 3, 9 patients (24%) IMS 2, and 8 patients (21%) IMS 1. Meanwhile, the DS was distributed as follows: 5 patients (13%) were assigned DS 5, no patients were assigned DS 4, 5 patients (13%) DS 3, 2 patients (5%) DS 2, and 26 patients (68%) DS 1.

For the 45 patients in the PET/MR cohort, the DS was inapplicable due to the absence of the mediastinal blood pool and liver. The IMS was distributed as follows: 5 patients (11%) were assigned to IMS 5, 9 patients (20%) to IMS 4, 12 patients (27%) to IMS 3, 9 patients (20%) to IMS 2, and 10 patients (22%) to IMS 1.

The kappa co-efficient for labeling the response assessment score on IMS and DS criteria were 0.910 and 0.958, respectively.

### Survival analysis of the PET/CT cohort

**Fig. 2:**
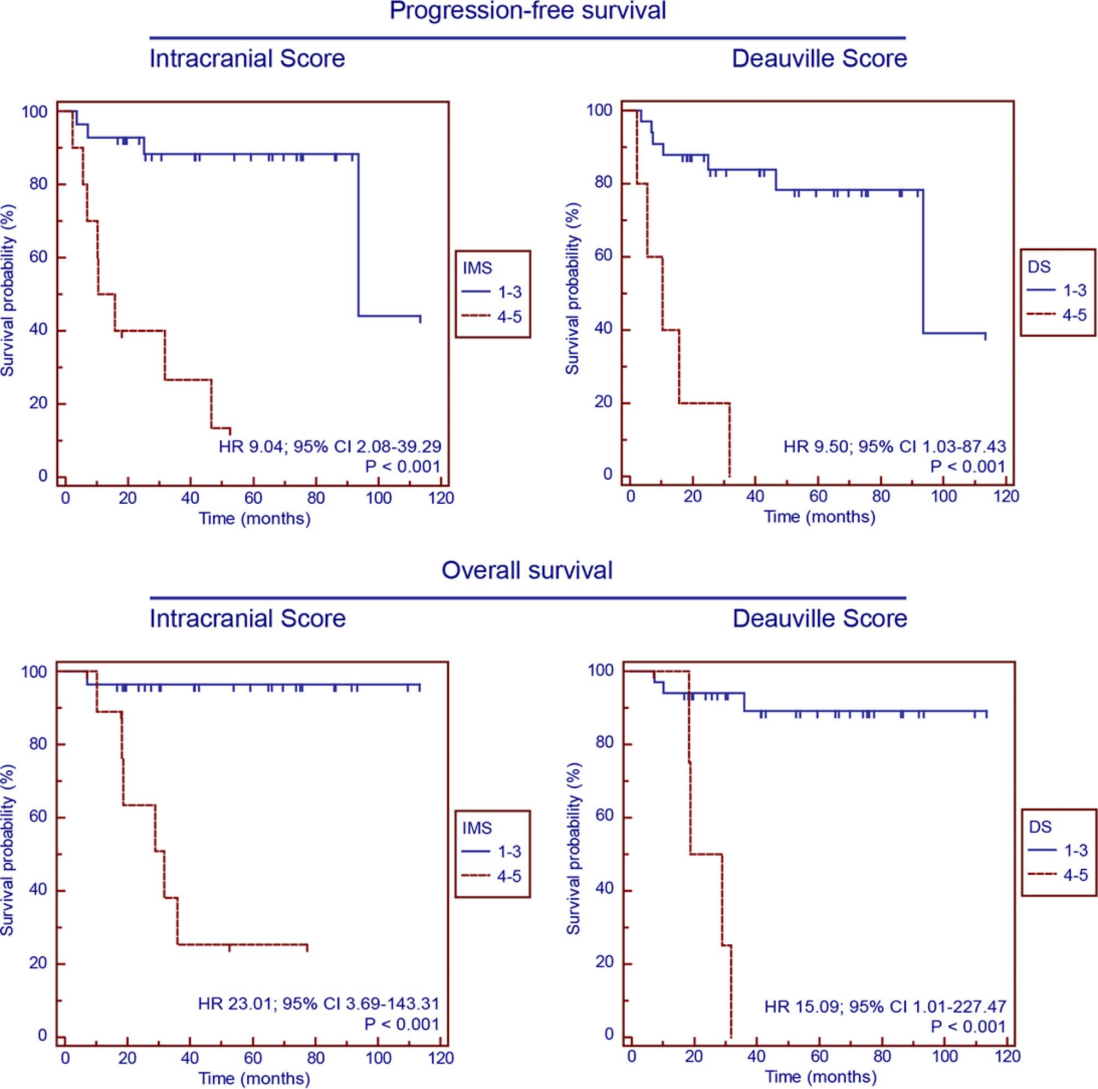
Survival analysis of PFS and OS in the PET/CT cohort according to the IMS and DS scales

Figure 2 and Supplementary Fig. 1 showed the Kaplan‒Meier survival curves for PFS and OS in the PET/CT cohort. After stratifying the patients into five distinct groups based on the IMS 1–5, significant disparities were observed among the groups in terms of PFS (*p* < 0.001) and OS (*p* < 0.001) survival rates. Further dichotomization of the patients according to the IMS into two groups (IMS 1–3 versus IMS 4–5 and IMS 1–4 versus IMS 5) revealed that the IMS 4–5 was associated with a markedly poorer prognosis than was the IMS 1–3 (PFS, HR 9.04, 95% CI 2.08–39.29, *p* < 0.001; OS, HR 23.01, 95% CI 3.69-143.31, *p* < 0.001), and the IMS 5 was linked to a significantly worse outcome than was the IMS 1–4 (PFS, HR 9.50, 95% CI 1.03–87.43, *p* < 0.001; OS, HR 15.09, 95% CI 1.01-227.47, *p* < 0.001). When the cohort was further divided into three groups (IMS 1–2, IMS 3–4 and IMS 5), it was found that an IMS 5 was associated with worse outcomes than both an IMS 1–2 and an IMS 3–4 (PFS, *p* < 0.001; OS, *p* < 0.001).

Significant differences in PFS and OS were observed among patients stratified into five groups based on the DS (*p* < 0.001; Supplementary Fig. 1). When patients were dichotomized using the DS (DS 1–3 versus DS 4–5 and DS 1–4 versus DS 5), DS 4–5 was associated with significantly worse outcomes than those with DS 1–3 (PFS, HR 9.50, 95% CI 1.03–87.43, *p* < 0.001); OS, HR 15.09, 95% CI 1.01-227.47, *p* < 0.001); and DS 5 was linked to significantly inferior outcomes than those with DS 1–4 (PFS, HR 9.50, 95% CI 1.03–87.43, *p* < 0.001; OS, HR 15.09, 95% CI 1.01-227.47, *p* < 0.001). When patients were divided into three groups (DS 1–2, DS 3–4 and DS 5), DS 5 was associated with worse PFS (*p* < 0.001) and OS (*p* < 0.001) than those with both DS 1–2 and DS 3–4.

As demonstrated in Table 2, Univariate and Multivariate Cox regression models were performed to evaluate the prognostic significance of clinical characteristics on PFS and OS, including age, gender, lesion number, site of tumor lesion, ECOG score, IELSG score, MSKCC score and PET results (DS and IMS). Multivariate analysis demonstrated the IMS (HR 9.04, 95% CI 2.08–39.29; *p* < 0.001) and DS (HR 9.50, 95% CI 1.03–87.43; *p* < 0.001) were the independent prognostic indicators for PFS in the PET/CT cohort. Similarly, for OS, the IMS (HR 18.34, 95% CI 2.15-156.67; *p* = 0.008) and DS (HR 15.18, 95% CI 2.68–85.92; *p* = 0.002) were identified as independent prognostic indicators in the PET/CT cohort (Table 2).

**Table 2:** Univariate and multivariate analysis for PFS and OS in PET/CT cohort of PCNSL patients

Variables	Univariate analysis			Multivariate analysis		
	HR	95% CI	*P*-value	HR	95% CI	*P*-value
**PFS**						
Age (≥ 60 vs. <60)	1.22	0.37–3.99	0.748			
Gender (Male vs. Female)	0.79	0.24–2.58	0.692			
Lesion number (Multiple vs. Single)	2.78	0.75–10.31	0.126			
Site of tumor lesion (Deep/both vs. Superficial)	1.61	0.75–3.47	0.224			
ECGO (2–3 vs. 0–1)	1.50	0.47–4.76	0.492			
IELSG (2–3 vs. 0–1)	1.73	0.55–5.45	0.351			
MSKCC (2 vs. 0–1)	1.35	0.73–2.49	0.346			
DS (4–5 vs. 1–3)	9.50	1.03–87.43	< 0.001^*^	(DS model) 9.50	1.03–87.43	< 0.001^*^
DS (5 vs. 1–4)	9.50	1.03–87.43	< 0.001^*^			
IMS (4–5 vs. 1–3)	9.04	2.08–39.29	< 0.001^*^	(IMS model) 9.04	2.08–39.29	< 0.001^*^
IMS (5 vs. 1–4)	9.50	1.03–87.43	< 0.001^*^			
**OS**						
Age (≥ 60 vs. <60)	0.53	0.10–2.71	0.442			
Gender (Male vs. Female)	1.33	0.30–5.97	0.707			
Lesion number (Multiple vs. Single)	6.38	0.77–53.20	0.087	(DS model) 4.22 (IMS model) 3.50	0.48–36.85 0.41–29.87	0.193 0.253
Site of tumor lesion (Deep/both vs. Superficial)	1.92	0.67–5.54	0.227			
ECGO (2–3 vs. 0–1)	0.91	0.20–4.07	0.898			
IELSG (2–3 vs. 0–1)	0.99	0.22–4.46	0.990			
MSKCC (2 vs. 0–1)	1.16	0.51–2.63	0.725			
DS (4–5 vs. 1–3)	15.09	1.01–227.47	< 0.001^*^	(DS model) 15.18	2.68–85.92	0.002^*^
DS (5 vs. 1–4)	15.09	1.01–227.47	< 0.001^*^			
IMS (4–5 vs. 1–3)	23.01	3.69–143.31	< 0.001^*^	(IMS model) 18.34	2.15–156.67	0.008^*^
IMS (5 vs. 1–4)	15.09	1.01–227.47	< 0.001^*^			

*Abbreviations* PCNSL, primary central nervous system lymphoma; PFS, progression-free survival; OS, overall survival; DS, Deauville 5-point score; IMS, intracranial metabolic score; 95% CI, 95% confidence interval. ^*^P < 0.05

### Survival analysis of the PET/MR cohort

**Fig. 3:**
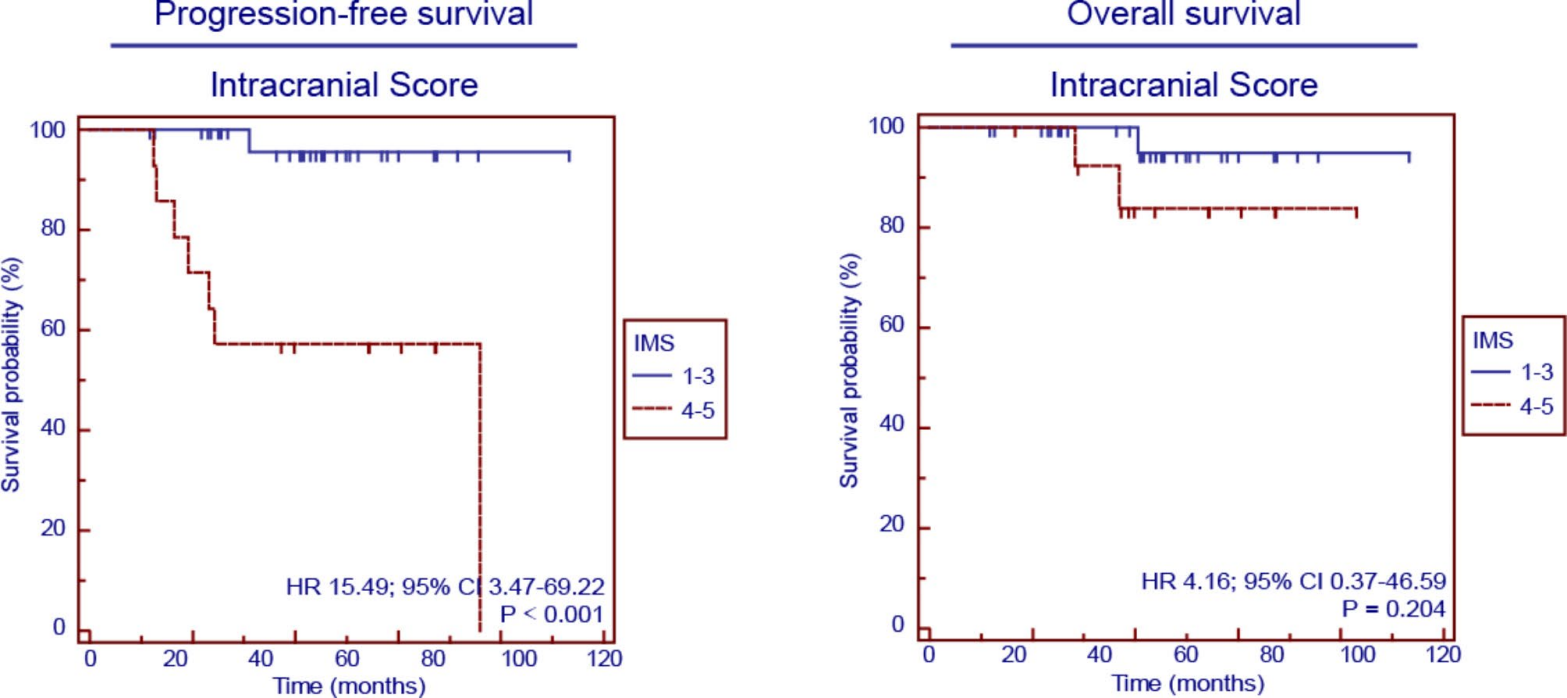
Survival analysis of PFS and OS in the PET/MR cohort according to the IMS scale

Figure 3 and Supplementary Fig. 2 shows the Kaplan‒Meier survival curves for PFS and OS in the PET/MR cohort. After stratifying the patients into five distinct groups based on the IMS 1–5, significant disparities were observed among the groups in terms of PFS (*p* < 0.001) survival. However, there was no significant difference between the 5 groups in OS (*p* = 0.440). Further dichotomization of the patients according to the IMS into two groups (IMS 1–3 versus IMS 4–5 and IMS 1–4 versus IMS 5) revealed that the IMS 4–5 was associated with markedly poorer PFS than was the IMS 1–3 (HR 15.49, 95% CI 3.47–69.22, *p* < 0.001), and the IMS 5 was linked to significantly worse PFS than was the IMS 1–4 (HR 35.47, 95% CI 1.25-1007.71, *p* < 0.001). However, there was no difference in OS between IMS 1–3 and IM 4–5 (HR 4.16, 95%CI 0.37–46.59, *p* = 0.204), or between IMS 1–4 and IMS 5 (HR 5.62, 95%CI 0.09-350.03, *p* = 0.109). When patients were categorized into three groups (IMS 1–2, IMS 3–4, and IMS 5), it was found that an IMS 5 was associated with shorter PFS than both an IMS 1–2 and an IMS 3–4 (*p* < 0.001). However, there was no difference in OS between these 3 groups (*p* = 0.154).

Similarly, univariate and Multivariate Cox regression models were also performed to evaluate the prognostic significance of clinical characteristics on PFS and OS, including age, gender, lesion number, site of tumor lesion, ECOG score, IELSG score, MSKCC score and IMS as showed in Table 3. Multivariate analysis revealed that the IMS (HR 15.49, 95% CI 3.47–69.22; *p* < 0.001) was an independent prognostic indicator for PFS in the PET/MR cohort. For OS, no independent prognostic indicator was identified in the PET/MR cohort (Table 3).

**Table 3:** Univariate and multivariate analysis for PFS and OS in PET/MR cohort of PCNSL patients

Variables	Univariate analysis			Multivariate analysis		
	HR	95% CI	*P*-value	HR	95% CI	*P*-value
**PFS**						
Age (≥ 60 vs. <60)	0.51	0.11–2.29	0.381			
Gender (Male vs. Female)	0.65	0.15–2.93	0.579			
Lesion number (Multiple vs. Single)	1.85	0.37–9.25	0.452			
Site of tumor lesion (Deep/both vs. Superficial)	5.46	0.12–245.32	0.382			
ECGO (2–3 vs. 0–1)	1.41	0.35–5.66	0.629			
IELSG (2–3 vs. 0–1)	2.93	0.35–24.38	0.319			
MSKCC (2 vs. 0–1)	0.63	0.22–1.84	0.402			
IMS (4–5 vs. 1–3)	15.49	3.47–69.22	< 0.001^*^	15.49	3.47–69.22	< 0.001^*^
IMS (5 vs. 1–4)	35.47	1.25–1007.71	< 0.001^*^			
**OS**						
Age (≥ 60 vs. <60)	Not available^**†**^		0.399			
Gender (Male vs. Female)	0.54	0.05–5.99	0.617			
Lesion number (Multiple vs. Single)	Not available^**†**^		0.486			
Site of tumor lesion (Deep/both vs. Superficial)	Not available^**†**^		0.599			
ECGO (2–3 vs. 0–1)	Not available^**†**^		0.435			
IELSG (2–3 vs. 0–1)	Not available^**†**^		0.479			
MSKCC (2 vs. 0–1)	0.19	0.00–78.82	0.586			
IMS (4–5 vs. 1–3)	4.16	0.37–46.59	0.204			
IMS (5 vs. 1–4)	5.62	0.09–350.03	0.109			

^*^P < 0.05; ^**†**^Unavailable HR value and wide range of 95% CI due to limited events in one group

### Predictive values of PET/CT and PET/MR according to assessment methods

**Fig. 4:**
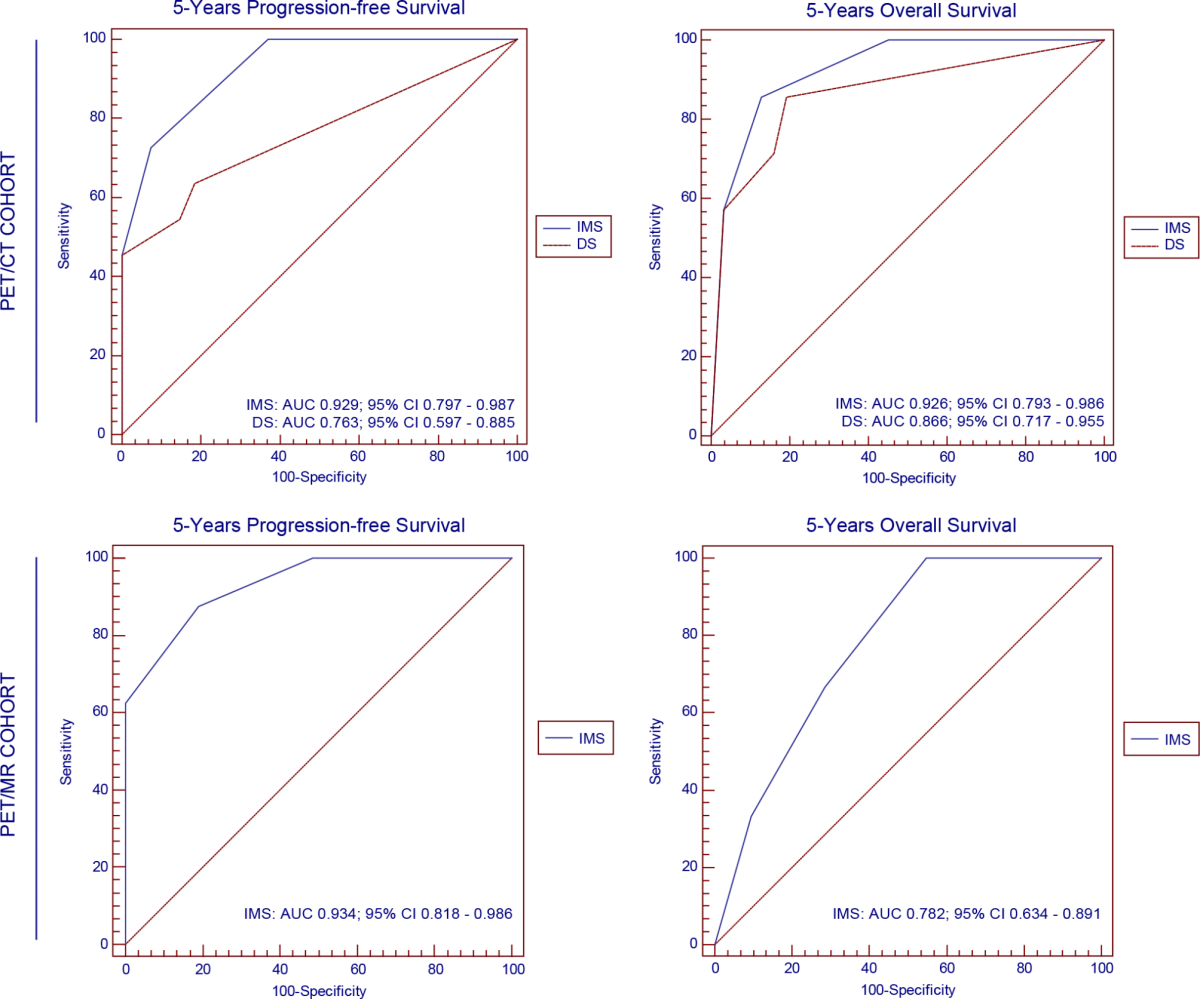
ROC curves of the IMS and DS in the PET/CT and PET/MR cohorts

ROC curves were constructed to evaluate the 5-year PFS and OS in patients from both the PET/CT and PET/MR cohorts, as depicted in Fig. 4. Within the PET/CT cohort, the AUC for the ability of the IMS to predict 5-year PFS in PCNSL patients was 0.929 (95% CI, 0.797–0.987; *p* < 0.001), surpassing the AUC of the DS scale of 0.763 (95% CI, 0.597–0.885; *p* <0.001). Similarly, the AUC for the IMS in forecasting 5-year OS was 0.926 (95% CI, 0.793–0.986; *p* < 0.001), which exceeded the AUC of the DS scale (0.866; 95% CI, 0.717–0.955; *p* = 0.003). In the PET/MR cohort, the AUCs for the IMS for predicting 5-year PFS and OS were 0.934 (95% CI, 0.818–0.986; *p* < 0.001) and 0.782 (95% CI, 0.634–0.891; *p* = 0.106), respectively.

In the PET/CT cohort, 33 (87%) patients were classified as negative category and 5 (13%) patients as positive if DS 4 and 5 were considered as positive. Given that DS 4 and 5 were considered as positive, the sensitivity, specificity, PPV, and NPV of EOT-PCT for PFS were 42%, 100%, 100% and 79%, respectively and for OS were 57%, 97%, 80% and 91%, respectively (Table 4). 10 (26%) patients were classified as positive category if IMS 4 and 5 were considered as positive, and 5 (13%) patients were classified as positive category if IMS 5 were considered as positive. Given that IMS 4 and 5 were considered as positive, the sensitivity, specificity, PPV, and NPV of EOT-PCT for PFS were 67%, 92%, 80% and 86%, respectively and for OS were 86%, 87%, 60% and 96%, respectively. Given that IMS 5 was considered as positive, the sensitivity, specificity, PPV, NPV of EOT-PCT for PFS were 42%, 100%, 100% and 79%, respectively and for OS were 57%, 97%, 80% and 91% (Table 4).

**Table 4:** Sensitivity, specificity, predictive values and AUC for outcomes

	PET/CT cohort				PET/MR cohort	
	DS 1,2,3 vs. DS 4, 5	DS 1,2,3,4 vs. DS 5	IMS 1,2,3 vs. IMS 4, 5	IMS 1,2,3,4 vs. IMS 5	IMS 1,2,3 vs. IMS 4, 5	IMS 1,2,3,4 vs. IMS 5
**PFS**						
Sensitivity (%)	41.7	41.7	66.7	41.7	87.5	62.5
Specificity (%)	100	100	92.3	100	81.1	100
PPV (%)	100	100	80.0	100	50.0	100
NPV (%)	78.8	78.8	85.7	78.8	96.8	92.5
AUC (95% CI)	0.708 (0.50-0.908)	0.708 (0.50-0.908)	0.795 (0.620–0.969)	0.708 (0.50-0.908)	0.843 (0.689–0.996)	0.813 (0.600-1.00)
**OS**						
Sensitivity (%)	57.1	57.1	85.7	57.1	66.7	33.3
Specificity (%)	96.8	96.8	87.1	96.8	71.4	90.5
PPV (%)	80.0	80.0	60.0	80.0	14.3	20.0
NPV (%)	90.9	90.9	96.4	90.9	96.8	92.5
AUC (95% CI)	0.770 (0.533-1.00)	0.770 (0.533-1.00)	0.864 (0.697-1.00)	0.770 (0.533-1.00)	0.690 (0.369-1.00)	0.619 (0.249–0.989)

*Abbreviations* AUC, area under the curve; PPV, positive predictive value; NPV, negative predictive value

In the PET/MR cohort, 14 (37%) patients were classified as positive category if IMS 4 and 5 were considered as positive, and 5 (13%) patients were classified as positive category if IMS 5 were considered as positive. Given that IMS 4 and 5 were considered as positive, the sensitivity, specificity, PPV, and NPV of EOT-PMR for PFS were 88%, 81%, 50% and 97%, respectively and for OS were 67%, 71%, 14% and 97%, respectively. Given that IMS 5 was considered as positive, the sensitivity, specificity, PPV, NPV of EOT PET for PFS were 63%, 100%, 100% and 92%, respectively and for OS were 33%, 91%, 20% and 93% (Table 4).

## Discussion

This study represents the first analysis of the prognostic value of EOT-PCT in PCNSL patients utilizing two criteria: the DS and the IMS. These findings suggest that the DS response assessment can be effectively applied to patients with PCNSL and that the IMS is a valuable assessment tool that surpasses the DS in terms of risk stratification at the end of treatment. Furthermore, the accuracy of the IMS was confirmed in a cohort of PCNSL patients who underwent the EOT-PMR.

Previous research has focused primarily on the diagnostic [22–25] and prognostic predictive value [11–16] of using pretreatment ^18^F-FDG PET/CT in PCNSL patients. However, few studies have explored the role of ^18^F-FDG PET/CT in evaluating the response to treatment for PCNSL [17, 18, 23, 26]. An initial small-scale study [26] involving 8 patients demonstrated that ^18^F-FDG PET conducted after the first cycle of chemotherapy or upon completion of chemotherapy might be able to predict complete remission post chemotherapy. Another small-scale study [23] involving 10 immunocompetent PCNSL patients also indicated that brain ^18^F-FDG PET could provide valuable information for the treatment of PCNSL, particularly in assessing treatment response. Recent research [18] with larger cohorts has corroborated these findings. Jo Jae-Cheol et al. [18] examined 66 PCNSL patients treated with MTX-based combination protocols to determine whether brain PET/CT could predict survival. They found that interim brain PET/CT was not associated with patient outcomes, but final PET/CT results held significant predictive value for PFS. However, these studies categorized PET results only as negative or positive, offering no guidance on how to use PET for evaluation in a clinical setting. In this study, we first applied the Deauville score to evaluate therapeutic efficacy in patients with PCNSL, which is widely used for systemic lymphoma [10, 27–29], and similar results were observed for PCNSL. In our study, patients with a DS of 4–5 had poorer outcomes than patients with a DS of 1–3.

PCNSL is a rare neoplasm that is typically confined to the brain parenchyma and eyes and infrequently affects the spinal cord [30]. Consequently, if the spinal cord is not implicated at the initial diagnosis, brain imaging is generally the primary focus for subsequent response evaluation in clinical practice. As our study demonstrated, the widely used DS can be applied to patients with PCNSL. However, its generalization in clinical applications may pose challenges. This is primarily because the assessment of DS depends on the uptake of hepatic and mediastinal blood pools. Unnecessary imaging of body parts outside of the head can negatively impact patient outcomes, escalate healthcare costs, and expose the patient to unnecessary ionizing radiation. Therefore, regional brain PET imaging is sufficient for clinical application, and there is an urgent need to establish a novel method based on FDG uptake in the brain.

In this study, we utilized a visual grading system based on the background of intracranial structures to analyze the prognostic value of EOT-PET imaging in PCNSL patients. The IMS is an extended version of the grading scale described by Meyer PT et al. [21]. Given the difference in FDG uptake between normal intracranial structures and the liver and mediastinal blood pools, as shown in Fig. 1b, we incorporated gray matter, white matter and cerebrospinal fluid as reference structures. Encouragingly, our PET/CT data indicated that the IMS is a valuable assessment scale for PCNSL that could outperform the DS in terms of risk stratification at the end of treatment. Furthermore, the feasibility of the IMS was validated in the PET/MR cohort, satisfactory risk stratification was found, and the IMS was identified as an independent prognostic indicator for worse OS in these patients.

Compared to patients with systemic non-Hodgkin lymphoma, those with PCNSL generally have a worse prognosis [3]. Up to 60% of patients who achieve complete remission according to the currently recommended method (IPCG criteria) will experience relapse within the first two years [31]. Therefore, an accurate end-of-treatment response evaluation is of paramount importance for PCNSL patients. The current standard response assessment for PCNSL imaging relies on the IPCG recommendations, which rely on MRI [6]. However, MRI often struggles to distinguish potential residual tumors from fibrotic scar tissue in stable tumors [23, 32]. Furthermore, the occurrence of early recurrence after treatment in patients with a complete response on MRI raises questions about the efficacy of MRI in assessing residual disease [31]. Although it was proved that ^18^F-FDG PET can reveal residual masses by distinguishing active from fibrotic tissue after chemotherapy in non-CNS Hodgkin lymphoma [33], its ability to visualize anatomical structures is limited. Taken together, combined brain PET/MR has emerged as the ideal modality for comprehensive imaging characterization of PCNSL, necessitating a metabolic evaluation system based on the background of intracranial structures. Recently, a prospective study included 65 immunocompetent PCNSL patients to assess the predictive and prognostic role of FDG PET/MR, which is the first to provide evidence supporting the role of PET/MR evaluation in predicting outcomes in PCNSL patients [34]. Encouragingly, our study further validated this result. The difference is that our research successfully developed and validated a novel scoring system capable of predicting outcomes.

At present, age and functional status at diagnosis are recognized as established prognostic factors [31], and the IELSG and MSKCC are the two multipoint scoring scales proposed for therapeutic trials [7, 20]. However, these methods are not sufficient for guiding the management of PCNSL. In our cohort, age, functional status, the IELSG score, and the MSKCC score were not identified as prognostic factors, which may be attributed to the sample size of our study. Recently, several studies have explored the role of pretreatment ^18^F-FDG PET as a prognostic indicator. A high SUV_max_ was associated with poorer survival in 17 PCNSL patients [12]. A visual scale correlating prognosis with the lesion SUV to normal cerebellar uptake was developed by Kasenda et al. [13]. The prognostic role of metabolic tumor volume (MTV) and total lesion glycolysis (TLG) has been emphasized [11, 15]. However, the application of these parameters in real clinical practice is still limited due to the lack of consensus and the limited number of patients included in these previous studies. In the present study, the DS and IMS were evaluated via end-of-treatment PET and were identified as dependent factors associated with prognosis. These results may aid in the prognostic stratification of patients who have achieved induction therapy. It should be highlighted that the decision regarding the end of a patient’s treatment is determined by a combination of factors, including the patient’s age, chemotherapy-related adverse effects, symptoms, and imaging findings.

This study is limited by its retrospective nature and the relatively small sample size, due to the disease’s rarity. However, this is currently the largest study investigating the role of ^18^F-FDG PET imaging in PCNSL. Another limitation is the high FDG uptake background in the brain. The high FDG uptake in normal gray matter may limit the clinical application of the CNS. Despite these limitations, ^18^F-FDG PET remains the most commonly used modality for CNS lymphoma. Furthermore, while high FDG uptake in gray matter may interfere with the detection of FDG-avid brain diseases, primary CNS lymphoma lesions are most commonly confined to the white matter, periventricular regions, and deep cerebral structures. This finding suggested that brain ^18^F-FDG PET may also be useful in this disease [14]. Third, a major criticism of PET/MR is the inability to accurately calculate SUV with MR based attenuation correction, so it is improper to put the direct comparison of SUV derived from MR based versus CT based attenuation maps. Several studies [35–37] addressed this concern showing good correlation between SUV derived from PET/CT and PET/MR devices, and PET/MRI showed equivalent performance to PET/CT in terms of qualitative results. Lastly, the scoring scale may not be applicable to lesions associated with leptomeningeal disease and eye involvement, which are diagnosed based on CSF flow cytometry and vitreous aspirate, as these lesions are usually not detectable via ^18^F-FDG PET [25]; moreover, these patients were excluded from this study. In fact, PCNSL patients are rarely affected by leptomeninges [38], and no patients exhibited leptomeningeal involvement in this study. Moreover, most immunocompetent patients with PCNSL present with a brain mass [39]. Therefore, the evaluation scale remains useful for the vast majority of PCNSL patients. Despite these limitations, this study provides novel insights for establishing a simple, practical, and accurate risk model for the prognosis of PCNSL patients.

Other PET tracers, such as ^11^C-Methionine [40], ^68^Ga-Pentixafor [41] and ^18^F-Fludarabine [42], with potentially high sensitivity and /or specificity are being explored for their usefulness in managing PCNSL. However, the available data on their utility is scarce, and further studies are required to definitively establish their impact on the management of this disease.

In conclusion, our study represents one of the largest investigations into response evaluation in PCNSL patients and is the first to apply brain PET/MR imaging at the end of induction chemotherapy. We discovered that EOT-PET, stratified by DS and the novel scoring system called the IMS, which is based on the background metabolism of intracranial structures, could effectively predict PFS and OS. The IMS may surpass the DS scale in prognostic accuracy. To substantiate this hypothesis, further prospective studies are warranted.

## Electronic supplementary material

Below is the link to the electronic supplementary material.

Supplementary Material 1

Supplementary Material 2

## Data Availability

No datasets were generated or analysed during the current study.

## Abbreviations

PCNSL: Primary central nervous system lymphoma
HD-MTX: High-dose methotrexate
ASCT: Autologous stem cell transplantation
WBRT: Whole-brain radiation
IPCG: International PCNSL Collaborative Group
MRI: Magnetic resonance imaging
CR: Complete response
PR: Partial response
FDG: Fluorodeoxyglucose
PET: Positron emission tomography
DS: Deauville 5-point score
IMS: Intracranial metabolism scoring
EOT: End-of-treatment
EOT-PCT: EOT-PET/CT
EOT-PMR: EOT-PET/MR
PFS: Prognostic free survival
OS: Overall survival
ECOG: Eastern Cooperative Oncology Group
KPS: Karnofsky score
LDH: Lactated hydrogenase
IELSG: International Extranodal Lymphoma Study Group
MSKCC: Memorial Sloan-Kettering Cancer Center
OSEM: Ordered subsets expectation maximization
PSF: Point spread function
DWI: Diffusion-weighted imaging
ADC: Apparent diffusion coefficient
FLAIR: Fluid attenuated inversion recovery
ROIs: Regions of interest
ROC: Receiver Operating Characteristic
AUC: Areas Under the Curve
SD: Standard deviation
95% CI: 95% confidence interval
HR: Hazard Rate
MTV: Metabolic tumor volume
TLG: Total lesion glycolysis
