# Supplementary figures and images for: Assessing the intracranial metabolic score as a novel prognostic tool in primary CNS lymphoma with end of induction-chemotherapy 18F-FDG PET/CT and PET/MR
Source: Cancer Imaging. 2024 Nov 11;24:152. doi: 10.1186/s40644-024-00798-1 (PMC11552111; doi:10.1186/s40644-024-00798-1)

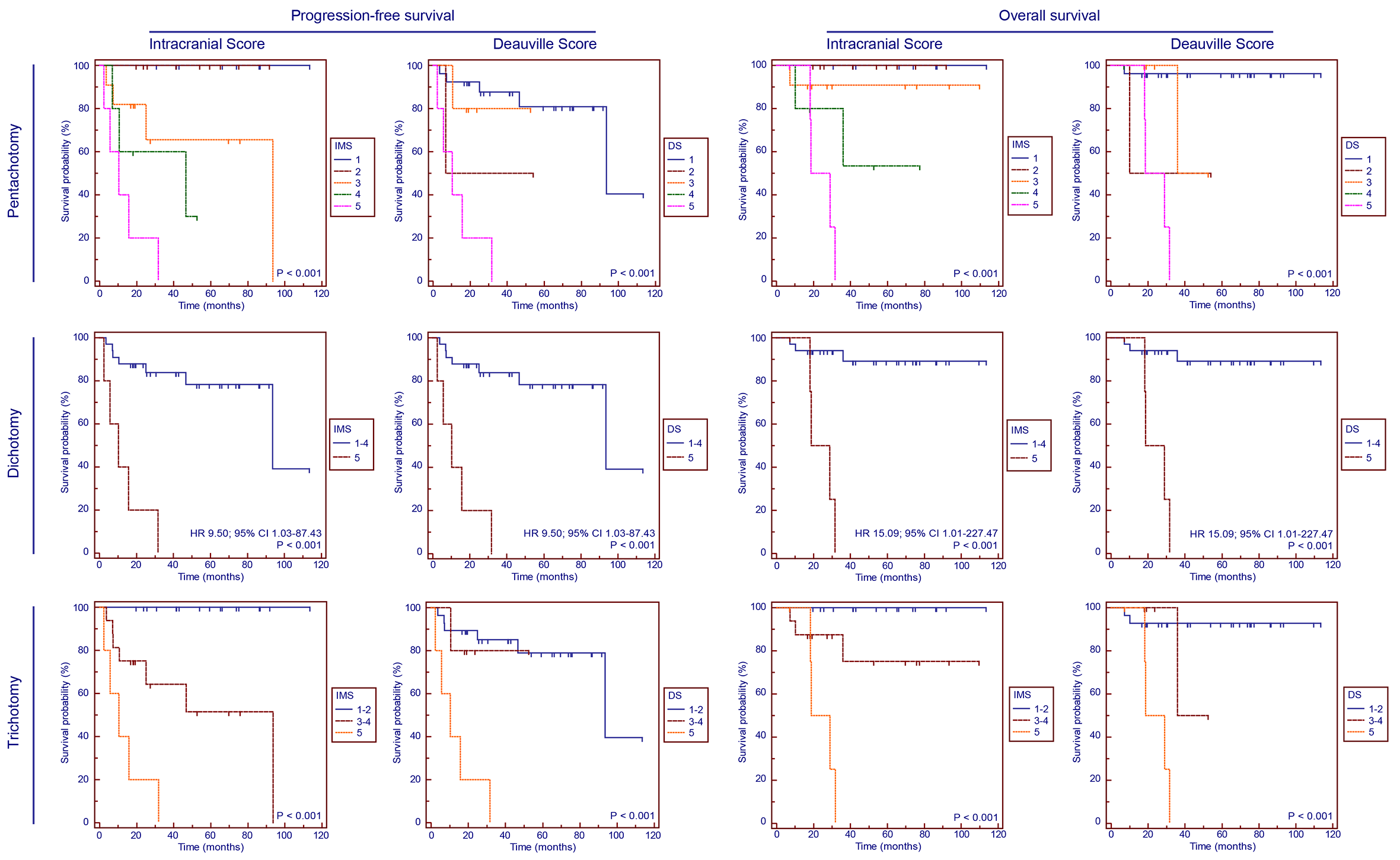

Supplement: Supplementary file 1 — Supplementary Material 1 [file 40644_2024_798_MOESM1_ESM.tif]

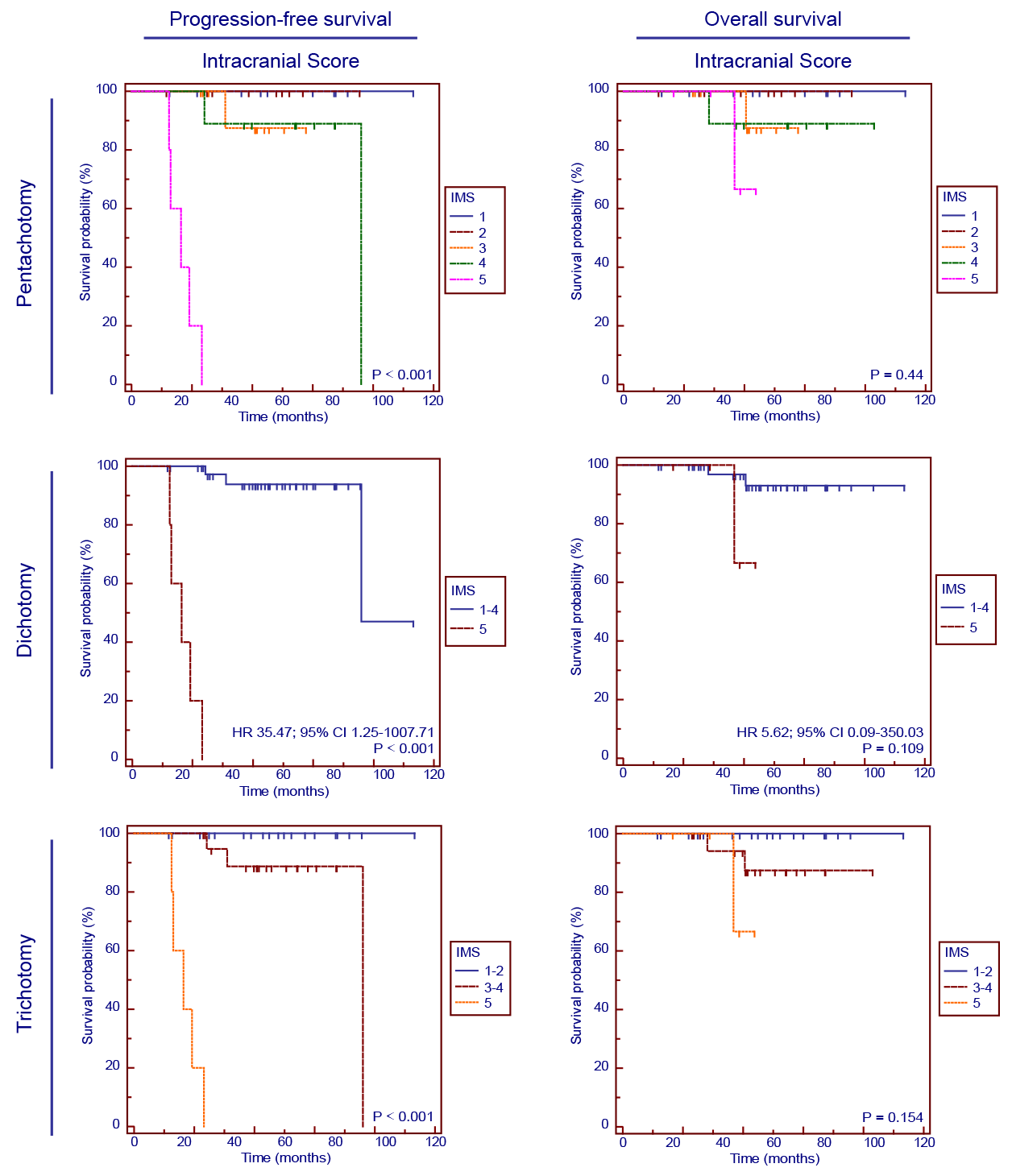

Supplement: Supplementary file 2 — Supplementary Material 2 [file 40644_2024_798_MOESM2_ESM.tif]
